# Quantification of spatiotemporal patterns of Ras isoform expression during development

**DOI:** 10.1038/srep41297

**Published:** 2017-01-24

**Authors:** Anna U. Newlaczyl, Judy M. Coulson, Ian A. Prior

**Affiliations:** 1 Division of Cellular and Molecular Physiology, Institute of Translational Medicine, University of Liverpool, L69 3BX, UK

## Abstract

Ras proteins are important signalling hubs frequently dysregulated in cancer and in a group of developmental disorders called Rasopathies. Three Ras genes encode four proteins that differentially contribute to these phenotypes. Using quantitative real-time PCR (qRT-PCR) we have measured the gene expression profiles of each of the Ras isoforms in a panel of mouse tissues derived from a full developmental time course spanning embryogenesis through to adulthood. In most tissues and developmental stages we observe a relative contribution of KRas4B > > NRas ≥ KRas4A > HRas to total Ras expression with KRas4B typically representing 60–99% of all Ras transcripts. KRas4A is the most dynamically regulated Ras isoform with significant up-regulation of expression observed pre-term in stomach, intestine, kidney and heart. The expression patterns assist interpretation of the essential role of KRas in development and the preponderance of KRas mutations in cancer.

Ras proteins are small molecular weight GTPases that act as molecular switches controlling key cell proliferation and anti-apoptosis pathways^1^. There are 3 ubiquitously expressed Ras genes encoding 4 proteins: HRas, KRas4A, KRas4B and NRas. Activating mutations in Ras isoforms are present in 20–30% of human cancer cases and in a group of developmental disorders known collectively as Rasopathies that affect as many as 1 in 1,000 people^2^,^3^.

Whilst the proteins encoded by the Ras genes are almost identical and with a common set of activators and effectors, they are not functionally redundant^4^. Most cancer types favour mutation of a specific Ras isoform, typically KRas, for reasons that are still unclear^3^. Isoform-specific biology is also seen amongst the Rasopathies where Noonan syndrome, associated with KRas and NRas, Cardiofaciocutaneous syndrome, associated with KRas and Costello syndrome, associated with HRas, exhibit overlapping but distinctive developmental and cardiovascular clinical characteristics^2^. Finally, knockout mouse studies reveal that only KRas is essential for mouse development^5^. Double NRas and HRas knockouts are viable and fertile whereas KRas knockouts die between E12.5 and birth exhibiting severe neuronal and cardiac abnormalities^5^,^6^.

ERas is a closely related Ras family member that is only expressed during mouse embryogenesis^7^. The gene is not generally expressed in humans but is aberrantly induced in gastric tumours^8^,^9^. Unlike the other Ras isoforms, ERas is constitutively active and binds preferentially to PtdIns 3-kinase (PI3K) but not Raf^10^. ERas knock-out mice were viable and without any obvious abnormalities; therefore, the precise contribution of ERas to early mouse development is still largely unknown^10^.

The relative contribution of Ras isoforms to total Ras signaling remains to be elucidated. The predominance of KRas mutations in cancer and the essential role of KRas in mouse development suggests that this is the most important isoform. This is complicated by the fact that KRas is alternatively spliced into two isoforms KRas4A and KRas4B that exhibit non-redundant contributions to tumourigenesis and development^11^,^12^. Furthermore, the compelling observation that KRas is the only isoform required for development was challenged by a study that substituted HRas into the KRas locus^13^. These mice survived to adulthood suggesting that the spatiotemporal expression profile of KRas was a dominant factor in the essential role of KRas for development. To help to interpret these data, it is important to know the relative expression profiles of all of the Ras isoforms.

To date, analysis of Ras isoform expression has either provided semi-quantitative results or focused on specific isoforms in a subset of adult tissues or cell lines^14^,^15^,^16^,^17^,^18^,^19^,^20^. An example is the Northern blot based analysis of HRas, KRas and NRas expression in mouse adult tissues and whole embryos^14^. This allowed comparison of Ras expression between tissues but the lack of calibrated standards precluded absolute quantitation of the relative contributions of each isoform to total Ras transcript abundance. Similarly, whilst Northern blot and quantitative real-time PCR (qRT-PCR) analysis has quantified the dominant cell and tissue expression of KRas4B versus KRas4A (typically representing ~60–99% of total KRas transcripts), the other Ras isoforms were not compared^18^,^19^.

We have used qRT-PCR to more precisely quantify the transcript copy number contribution for each of the Ras isoforms to total Ras expression across the major tissues and through a full developmental time course. Ras transcripts are highly abundant in all tissues with the highest expression seen in brain where Ras biology is virtually uncharacterized. The dominant contribution of KRas4B to total Ras expression may explain the major role of this isoform in cancer and development whilst the ubiquitous and highly dynamic pattern of KRas4A expression suggests novel biology for this poorly studied isoform.

## Results

### Optimising qRT-PCR for Ras isoforms

**Figure 1:**
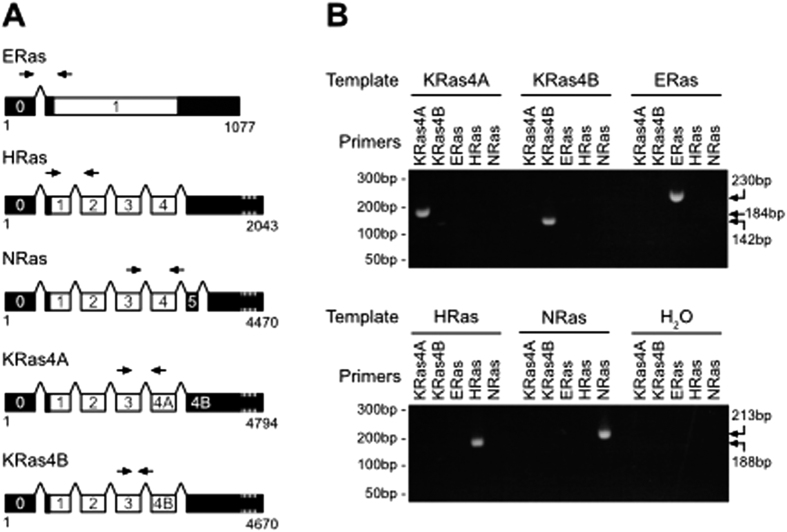
Specific PCR amplification of Ras isoforms. (**A**) Schematic of exon organization of Ras isoforms with coding areas in white and non-coding in black. Arrows indicate locations of isoform-specific Ras primer pairs. For KRas4B, the reverse primer was designed to span the exon3/4B splice boundary. (**B**) Ras isoform-specific primers were used in end-point PCR reactions with the indicated linearised templates of each Ras isoform plasmid. Products of the correct size were observed and there was no evidence of cross-amplification.

Accurate measurement of Ras isoform expression using quantitative PCR necessitated the design and optimisation of PCR primers that could specifically amplify each of the Ras isoforms. During splicing of KRas, the difference between KRas4A and KRas4B is the presence or absence of the 4A exon. Since exon 4B is present in the untranslated region of KRas4A mRNA, we developed a reverse primer covering the exon 3/4B splice junction that would specifically amplify KRas4B (Fig. 1A). Standard end-point PCR followed by agarose gel electrophoresis confirmed that single products of the correct size were amplified by each Ras primer pair from the cognate Ras plasmid (Fig. 1B). No cross-amplification was seen with other Ras isoforms for any of the isoform-specific primer pairs. These primer pairs were also able to specifically amplify bands of the correct size from cDNA (Supplementary Figure 1A). No primer dimers were observed under optimised qRT-PCR conditions used for subsequent tissue analysis (Supplementary Figure 1B). We did not identify any reference transcripts that were suitable for comparison across tissues and through development. We therefore generated standard curves for each isoform-specific PCR reaction such that we could then determine absolute copy number for each template as a function of the threshold cycle (Ct) value (Supplementary Figure 1C). These were used to determine transcript copy number in subsequent tissue analysis. We note that there may be differences in the amplification efficiency of plasmid DNA versus cDNA that may influence the final transcript copy number values that we obtain. However, in contrast to supercoiled plasmid, amplification of linearised plasmid (as used here) has been shown to give similar quantitation to amplification of cDNA^21^.

### KRas4B expression is significantly higher than the other Ras isoforms

**Figure 2:**
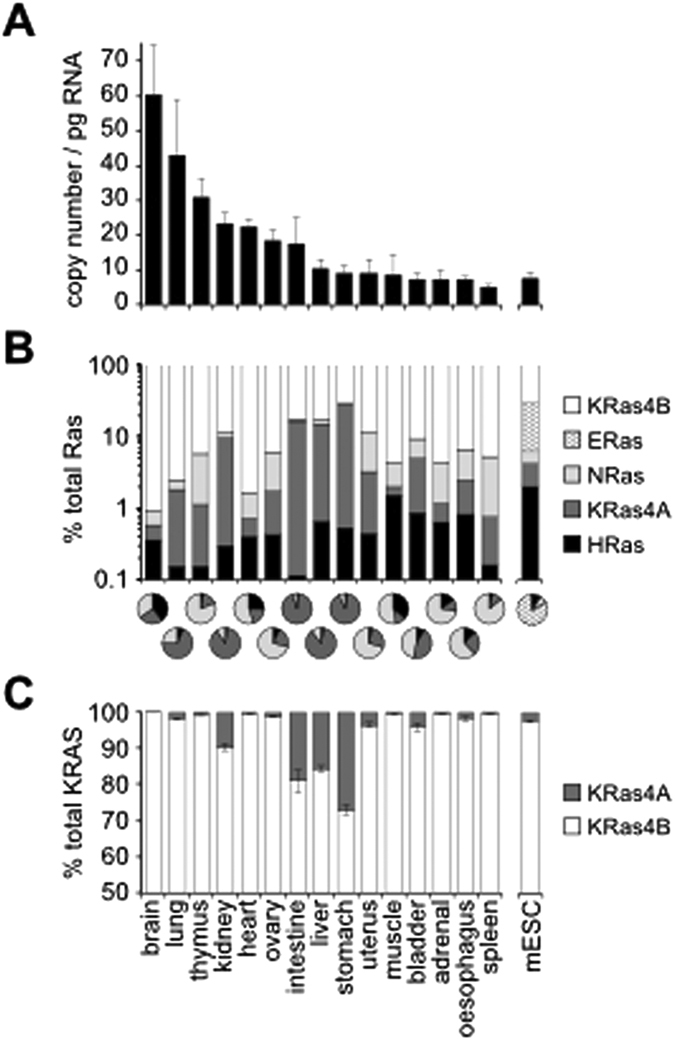
Quantitation of Ras isoform expression in adult tissues. (**A**) Total Ras expression summed from qRT-PCR quantitation of HRas, KRas4A, KRas4B, NRas and ERas. (**B**) Analysis of the relative expression of Ras isoforms indicates that KRas4B represents 70–99% of total tissue Ras mRNA transcripts. Histogram employs log scale to show contribution of minor isoforms. Pie charts show the relative expression of all Ras isoforms except KRas4B. (**C**) Contribution of KRas4A and KRas4B to total KRas expression. n = 3 adult mice per tissue, n = 3 R1 mESC cell samples.

We chose the outbred CD-1 mouse strain to study expression of Ras isoforms in normal tissues. Analogous to the case in humans, each of the Ras genes are present as 2 alleles in mice^22^. qRT-PCR analysis of 15 adult mouse tissues revealed a range of total Ras expression from 5–60 copies per pg of RNA (Fig. 2A, Supplementary Table 1). Since the average cell contains ~10 pg of RNA^23^,^24^, this translates into approximately 50–600 copies per cell making total Ras transcripts highly abundant compared to most other gene transcripts^25^. In every tissue studied KRas4B was the most abundant isoform representing 70–99% of total mRNA copies (Fig. 2B). In most tissues, NRas was the next most abundant isoform except for a tissue subset of stomach, intestine, lung, liver and kidney where significant KRas4A expression was observed. Expression of both of these isoforms ranged from 0.1–1 copy per pg of RNA in most tissues (Supplementary Table 1). HRas was consistently the least abundant isoform, present at <0.1 copies per pg of RNA (~1 mRNA copy per cell) in all tissues except brain and muscle (Fig. 2B). For comparison, we analysed Ras abundance in undifferentiated R1 mouse embryonic stem cells (mESCs). Whilst KRas4B again represented the most abundant isoform, the early embryo-specific ERas accounted for ~20% of total Ras transcripts. Notably, KRas4A expression in mESCs represents <0.005% total Ras transcripts with fewer than 0.04 molecules per pg of RNA (<0.5 copies per cell) suggesting little if any contribution to early development.

Splicing of KRas into KRas4A and KRas4B isoforms is of interest since both isoforms will harbour the oncogenic mutations observed in ~20% of human cancers and have differential cancer promoting activities^11^. KRas is frequently mutated in lung and intestinal cancers and both exhibit higher than average KRas4A expression (Supplementary Table 1) with KRas4A also contributing a higher share of total KRas expression in intestine (Fig. 2C). Whilst previous qRT-PCR-based analysis of human adult tissues has suggested that KRas4A is not ubiquitiously expressed^17^, we detected KRas4A in every tissue examined; albeit at low levels and in most cases representing <5% of total KRas transcripts.

### Spatiotemporal expression profile of Ras isoforms during development

**Figure 3:**
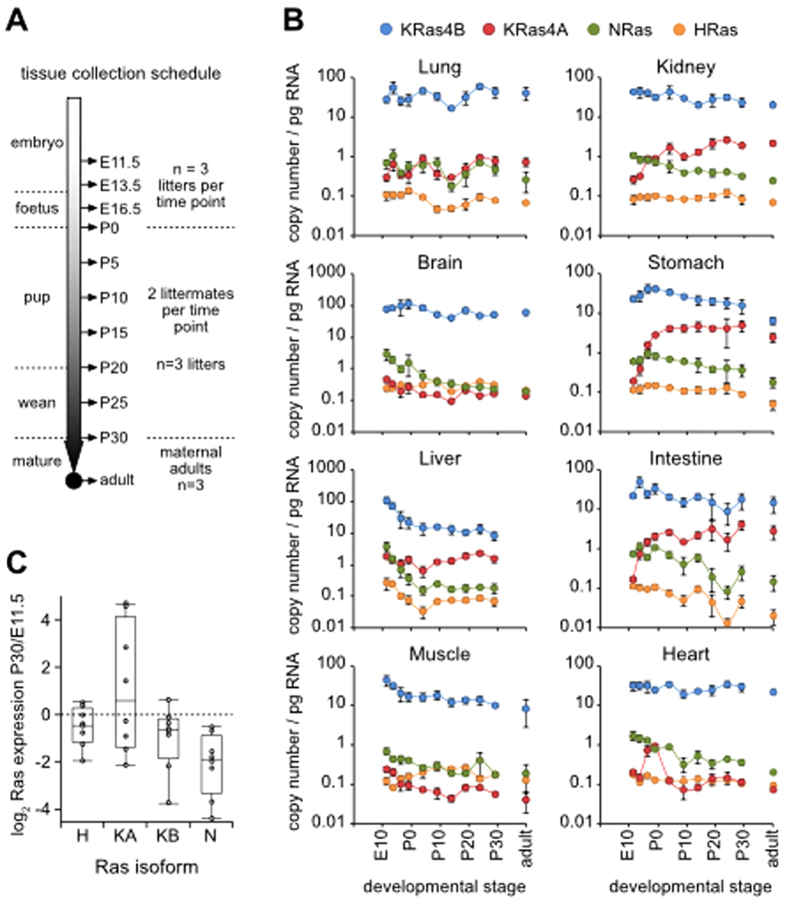
Ras isoform expression during development. (**A**) Schematic of tissue collection schedule during developmental stages. (**B**) qRT-PCR quantitation of Ras isoform expression in mouse tissues. (**C**) Comparison of expression at P30 with E11.5 reveals dynamic changes amongst all Ras isoforms across tissues with most showing a decrease in Ras expression during maturation. Tukey plots depict data minima and maxima, the range between the first and third quartiles and the line indicates the median.

We quantified the expression of Ras isoforms during each of the key stages in development from the embryo to the mature adult (Fig. 3A). Given the small size of the nascent organs during embryogenesis, we pooled organs from whole litters (8–14 individuals) for each time point for each biological repeat pre-birth. For neonates, we used two littermates for each time point which, together with the maternal adult, generated a post-birth time-course of related individuals for each biological replicate. Pre-birth, whole organs were pooled for analysis; post-birth, we randomly sampled each of the organs with the exception of brain and intestine where cerebral cortex and small intestine were selectively harvested.

Contrasting patterns of Ras expression can be seen between isoforms and tissues during development. Whilst most tissues harbour at least one Ras isoform exhibiting dynamic patterns of expression, the expression of all four Ras isoforms in lung remains stable throughout the ~40 day time course (Fig. 3B). Here again, KRas4B is the most abundant transcript at each time point throughout development. The largest changes can be seen with KRas4A that increases 10–25-fold over the developmental time-course in kidney, stomach and intestine. Notably, a spike in expression around birth can also be seen in heart; a tissue where KRas4A has not previously been thought to be expressed^17^. For the other isoforms there is a general trend for either stable expression or a decrease in expression over time that is most clearly seen with NRas (Fig. 3C). Together, this represents the most comprehensive analysis of Ras isoform expression during development available, with absolute quantitation that enables relative contributions of Ras isoforms to total Ras transcript expression to be accurately determined.

## Discussion

We have generated the first comparative analysis of Ras isoform gene expression in major tissues and at key developmental stages. In most tissues we observed a relative contribution of KRas4B > > NRas ≥ KRas4A > HRas to total Ras expression. The dominance of KRas4B expression (60–99% of Ras transcripts) in all tissues throughout all developmental stages analysed may explain the essential role of this isoform for viability and normal development. When considering the gene expression patterns it’s important to note that our data represent gross tissue measurements and do not account for sub-anatomical differences in Ras expression that may exist due to the heterogeneous cell populations present within each tissue. Similarly, we did not systematically analyse functional sub-regions of tissues such as the brain to explore whether the expression patterns that we observed in the cerebral cortex were observed in other sub-regions.

Whilst gene expression levels do not provide mechanistic insight into Ras function, they remind us of some relatively neglected areas of Ras biology. For example, the highest levels of Ras expression were observed in the brain where KRas isoforms represent >99% of Ras transcripts (Fig. 2). Although we do not know the relative contributions of neurons or associated glial cells to these values, the function of Ras in the central nervous system is enigmatic. KRas knockout mice and the Costello and Noonan syndrome Rasopathies exhibit brain stem and neurodevelopmental defects indicating a requirement for Ras in normal brain development^2^,^5^. This may be linked to the promotion of brain stem cell proliferation by KRas^26^; however, in terminally differentiated, non-proliferating neurons this functionality become superfluous. Instead, accumulating evidence suggest an important alternative signaling function for Ras in regulating synaptic plasticity and learning^27^,^28^,^29^,^30^,^31^.

Another under-explored area of Ras biology is the function of KRas4A. Whilst KRas4B, HRas and NRas are ubiquitously expressed, KRas4A is generally thought to have a more restricted expression profile based on human tissue analysis^17^. In contrast, in our analysis and a previous Northern blotting based screen of KRas4A expression it was detected in all mouse tissues studied including those where is was not observed in human samples (Fig. 2; ref. 16). Our data describing a bias towards KRas4B splicing is consistent with previous observations that KRas4B represents the majority of total KRas gene expression^18^,^19^. For example, in a recent qRT-PCR-based study by the Mark Philips laboratory, KRAS4A was expressed at varying levels across cancer cell lines derived from several tissue types and represented ≤25% of total KRAS expression in 25 of the 30 cell lines studied^18^. Furthermore, KRAS4A represented 15–50% of total KRAS expression in 17 human colorectal cancer samples indicating highly context-dependent regulation of KRAS splicing^18^.

The reversible post-translational modifications that direct membrane localization and define Ras isoforms mean that KRAS4A may have the capacity to behave like KRAS4B or NRAS dependent on whether or not it is palmitoylated^32^. Although this does not mean that KRas4A can substitute for the essential mouse developmental role of KRAS4B^12^, the expression of KRAS4A was the most dynamically regulated of any of the Ras isoforms during development (Fig. 3). The transient increase in KRas4A expression in the heart around birth coincides with the time when cardiac myocytes exit the cell cycle and undergo hypertrophy^33^. The ~10–25-fold increase in KRas4A expression in stomach and intestine between E11.5 and birth occurs when the gut elongates, differentiates into a stratified epithelium and the intestinal crypt and villus structure develops^34^. Nothing is known about the possible role of KRas4A in these processes; however, the gut developmental programme that includes Wnt/β-catenin signaling is frequently dysregulated in colorectal cancer where KRas mutations are also typically found^3^,^35^.

The preponderance of KRas mutations in cancer has long been known but is still not properly understood^36^. KRas mutations are frequently observed in lung, colon and pancreas and this is proposed to be due to the ability of KRas to promote endodermal stem cell expansion^37^. More recently, an alternative model suggested that the relative expression levels of the Ras isoforms are an important factor. KRas contains a higher proportion of rare codons compared to the other isoforms and this limits the translation efficiency of this isoform^38^. Optimising the codons resulted in higher KRas protein expression and a reduced tumour burden consistent with previous studies that showed that high levels of Ras expression promote senescence rather than tumourigenesis^39^,^40^. These observations lead to the proposal that rare codon restricted KRas protein expression in comparison with the other isoforms contributes to the extra oncogenic potency of KRas.

The notion that KRas protein expression is limited contrasts with quantitation of Ras isoform protein abundance in cancer cell lines where KRas was the most abundant isoform in the majority of cell lines^15^,^41^. Our analysis of Ras isoform gene expression in normal tissues is broadly consistent with the dominance of KRas protein expression in cancer cell lines representing a wide range of tissues; however, the relative proportions are significantly different. Whilst in cell lines KRas typically represented 40–60% of total Ras protein abundance, we find the KRas contributes ~60–99% of total Ras transcript copies (Fig. 2). The high KRas transcript copy number may represent a compensatory mechanism for overcoming rare codon influence on translation. This is likely to be context dependent since transfer RNA composition varies between tissues^42^,^43^.

Whilst we have not measured protein expression, the relative concentrations of Ras proteins within the cell are likely to be a predominant factor regulating isoform-specific behavior. It’s tempting to speculate that the reason why KRAS is the most important isoform during development and in cancer is because a high KRAS expression level versus the other Ras isoforms means that Ras networks more often than not initiated by KRAS and therefore tuned to respond to this isoform. Clearly, gene expression levels do not necessarily correlate to protein abundance and absolute quantitation of Ras protein abundance in tissues is now required to determine if and why the mRNA levels of Ras isoforms are not proportional to the protein levels in tissues. This will then enable a detailed analysis of the potential post-transcriptional or translational regulatory mechanisms that may be responsible for any difference and an assessment of the role of rare codons in regulating KRAS expression levels and oncogenic potential.

In summary, we have quantified the transcript expression profiles of each of the Ras isoforms through a full developmental cycle from the emergence of major organs through to adulthood. The spatio-temporal patterns of expression highlight neglected areas of Ras biology and inform models that try to explain the essential role of KRas in development and the preponderance of KRas mutations in cancer.

## Methods

### Cell culture

R1 mouse embryonic stem cells^44^ were cultured on a feeder layer of mouse STO fibroblast cells^45^ in high glucose DMEM containing 15% FBS, 1% L-glutamine, 0.1 mM non-essential amino acids, 0.01% mercaptoethanol, 260 U/ml Leukemia Inhibitory Factor (LIF). To separate undifferentiated R1 ESCs from STO cells, the cells were trypsinised, replated onto dishes coated with 0.1% gelatin and allowed to adhere for 20 minutes. The rapid adherence of STO cells versus R1 ESCs results in a non-adherent fraction highly enriched with R1 ESCs that were used for RNA extraction.

### Tissue collection

Schedule 1 euthanasia of mice and all associated procedures and protocols were carried out in accordance with Home Office guidelines and regulations. All methods and protocols were approved by the University of Liverpool Animal Welfare and Ethical Review Body. Tissue from euthanised embryos, neonates and maternal adults of CD-1 mice (Charles River) were harvested at the indicated developmental time points, carefully rinsed in cold PBS and snap frozen in liquid nitrogen. Whole organs from all littermates were pooled for pre-birth time points. Post-birth, sections of organs were randomly sampled with the exception of brain (cerebral cortex), intestine (small intestine) and muscle (hind leg skeletal muscle). Care was taken to wash away the associated blood and contents of heart, stomach and intestine derived from neonates and adults before snap freezing and storage at −80 °C.

### Quantitative RT-PCR

RNA was isolated from cultured cells or fresh frozen tissue samples using the RNA/Protein Purification kit (Norgen). 20–50 mg of thawed tissue was homogenised in RNA Lysis buffer (Norgen) using a PowerLyzer (MO BIO Laboratories), transferred to RNAse free microfuge tubes and debris pelleted before RNA extraction. Reverse transcription was performed on 1 μg total RNA using RevertAid H-minus M-MuLV reverse transcriptase (Fermentas) and an oligodT primer (Promega); control reactions (-RT) omitted reverse transcriptase. Quantitative real-time RT-PCR (qRT-PCR) was performed in triplicate (n = 3 biological repeats each comprising 2 technical replicates) using SYBR Green Supermix (Bio-Rad) on a CFX Connect Thermal Cycler (Bio-Rad). The following primers designed to generate isoform-specific Ras amplicons were used: ERas forward: 5′-GGTCAGATCCGCCTACTGCC-3′, reverse: 5′-CACCACCACTGCCTTGTACTCG-3′; HRas forward: 5′-GCTGTAGAAGCTATGACAGAATAC-3′, reverse: 5′-GCTGTGTCTAAGATGTCCAGTAG-3′; KRas4A forward: 5′-AGATGTGCCTATGGTCCTGGTAG-3′, reverse: 5′-CAATCTGTACTGTCGGATCTCTCTC-3′; KRas4B forward: 5′-GATGTGCCTATGGTCCTGGTAG, reverse: 5′-CATCGTCAACACCCTGTCTTG-3′; NRas forward: 5′-CAAGGACAGTTGACACAAAGC-3′, reverse: 5′-TGTCTTACTACATCAGCACACAG-3′. Samples were amplified in a three step reaction with annealing at 62 °C (30 s); melt curves were analysed after 40 cycles. Results were normalised to standard curves generated from 5-fold dilution series of the following linearised full-length mouse Ras isoform plasmids: pCR4-TOPO-ERas (IMAGE ID: 8734026), pCR-Blunt II-TOPO-KRas-4A (IMAGE ID: 40052024) and pCMV-SPORT6-KRas4B (IMAGE ID: 3158212) (Source BioScience). Full-length mouse HRas and an NRas fragment for standard curve generation were amplified from mouse R1 ESCs using the following primers: forward: 5′-GAGATCTCTATGACAGAATACAAGCTTG-3′, reverse: 5′-ACCTCGAGATCAGGACAGCAC-3′ and NRas forward: 5′-CAAGGACAGTTGACACAAAGC-3′, reverse: 5′-TGTCTTACTACATCAGCACACAG-3′ and sub-cloned into pCR4-TOPO. A full-length pCMV-SPORT6-NRas plasmid (IMAGE ID: 6475312) (Source BioScience) was used to check for non-specific cross amplification.

## Additional Information

**How to cite this article:** Newlaczyl, A. U. *et al*. Quantification of spatiotemporal patterns of Ras isoform expression during development. *Sci. Rep.* **7**, 41297; doi: 10.1038/srep41297 (2017).

**Publisher's note:** Springer Nature remains neutral with regard to jurisdictional claims in published maps and institutional affiliations.

## Supplementary Material

Supplementary Figure 1

Supplementary Table 1
