# Supplementary material for: Quantification of spatiotemporal patterns of Ras isoform expression during development
Source: Sci Rep. 2017 Jan 24;7:41297. doi: 10.1038/srep41297 (PMC5259795; doi:10.1038/srep41297)
Supplement: Supplementary Figure 1 [file srep41297-s1.pdf]

Quantification of spatiotemporal patterns of Ras isoform expression during development

Anna U. Newlaczyl[1], Judy M. Coulson[1,2] and Ian A. Prior[1,2]

Supplementary Data

[1] Division of Cellular and Molecular Physiology, Institute of Translational Medicine, University of Liverpool, L69 3BX, UK.

[2] Corresponding Author: Ian A. Prior
Email: iprior@liverpool.ac.uk
Tel: +44-151-794-5332
Fax: +44-151-794-4434

[2] Corresponding Author: Judy M. Coulson
Email: jcoulson@liverpool.ac.uk
Tel: +44-151-794-5850
Fax: +44-151-794-4434

Supplementary Table 1. *Ras isoform tissue expression profiles.* Transcript copy number in adult tissues and a ten point developmental time course.

Supplementary Figure 1. *Specific qRT-PCR amplification and quantitation of Ras isoforms.* (A) The correct sized products of Ras isoforms are specifically amplified from mESC cDNA using end-point qRT-PCR; no products are seen in the minus reverse transcriptase (-RT) and dH_2O negative controls. (B) Melt curve analysis of qRT-PCR amplification products from each tissue for each Ras isoform show no evidence of primer dimer; melt curve for amplification from plasmid template shown in red. (C) qPCR analysis of 5-fold dilution series of plasmids for each of the Ras isoforms generated standard curves allowing conversion of Ct measurements into copy number values for absolute quantitation of Ras isoform transcript expression in tissues.

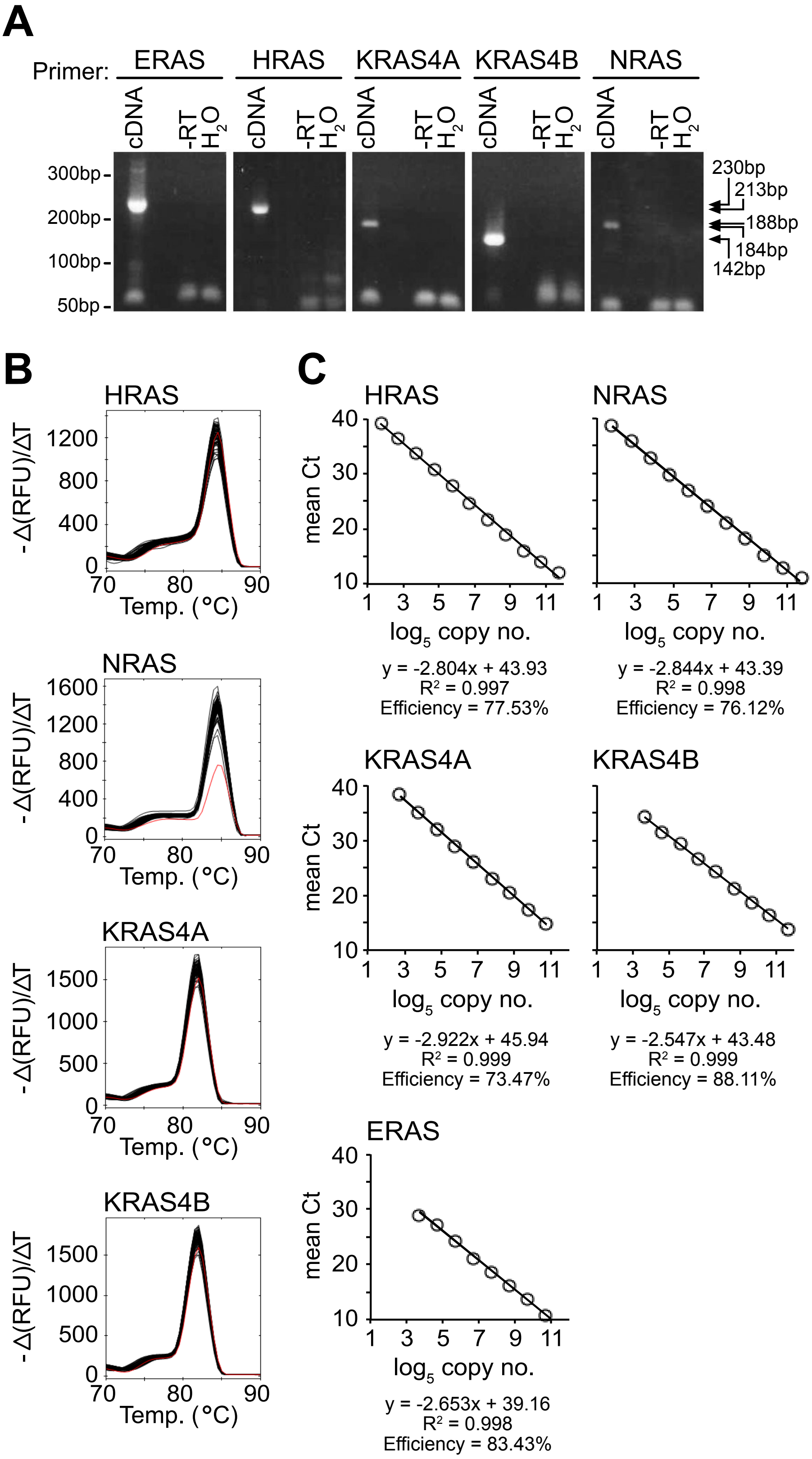
A
Primer:
ERAS
HRAS
KRAS4A
KRAS4B
NRAS
cDNA
-RT
H2O
300bp
200bp
100bp
50bp
230bp
213bp
188bp
184bp
142bp
B
HRAS
NRAS
KRAS4A
KRAS4B
-Δ(RFU)/ΔT
Temp. (°C)
C
HRAS
NRAS
KRAS4A
KRAS4B
ERAS
mean Ct
log5 copy no.
y = -2.804x + 43.93
R2 = 0.997
Efficiency = 77.53%
y = -2.844x + 43.39
R2 = 0.998
Efficiency = 76.12%
y = -2.922x + 45.94
R2 = 0.999
Efficiency = 73.47%
y = -2.547x + 43.48
R2 = 0.999
Efficiency = 88.11%
y = -2.653x + 39.16
R2 = 0.998
Efficiency = 83.43%
